# Construction of eGFP-Tagged Senecavirus A for Facilitating Virus Neutralization Test and Antiviral Assay

**DOI:** 10.3390/v12030283

**Published:** 2020-03-05

**Authors:** Fuxiao Liu, Yilan Huang, Qianqian Wang, Hu Shan

**Affiliations:** 1 College of Veterinary Medicine, Qingdao Agricultural University, Qingdao 266109, China; hyl1025@163.com (Y.H.); wqq107323@163.com (Q.W.); 2 Qingdao Research Center for Veterinary Biological Engineering and Technology, Qingdao 266109, China; 3 Shandong Collaborative Innovation Center for Development of New Veterinary Pharmaceuticals, Qingdao 266109, China

**Keywords:** Senecavirus A, reverse genetics, eGFP, virus neutralization test, ribavirin, apigenin

## Abstract

Senecavirus A (SVA), also known as Seneca Valley virus, is an emerging virus that causes vesicular disease in pigs. This virus belongs to the genus *Senecavirus* in the family *Picornaviridae*. The SVA CH-LX-01-2016 was isolated from Guangdong Province of China in 2016. In this study, a recombinant SVA CH-LX-01-2016 was constructed using reverse genetics, and proven to be able to express efficiently an enhanced green fluorescent protein (eGFP) in vitro. This eGFP-tagged recombinant SVA (rSVA-eGFP) exhibited a high capacity for viral replication. Its fluorescence-tracked characteristics greatly facilitated both virus neutralization test (VNT) and antiviral assay. The rSVA-eGFP-based VNT was used to detect eight porcine serum samples, out of which four were determined to be neutralization titer-positive. Subsequently, two antiviral drugs, ribavirin and apigenin, were assayed for evaluating both effects against the rSVA-eGFP in vitro. The result showed that only the ribavirin exhibited an anti-SVA activity.

## 1. Introduction

Senecavirus A (SVA), previously known as Seneca Valley virus, is an emerging pathogen in China [1,2]. It is a non-enveloped, positive-sense and single-stranded RNA virus, and classified into the genus *Senecavirus* in the family *Picornaviridae*. The SVA was incidentally isolated from the PER. C6 (transformed fetal retinoblast) cell line in 2002, and had evidently been introduced into the cell culture through the use of contaminated fetal bovine serum or porcine trypsin [3]. SVA infection is characterized by porcine vesicular lesions on coronary bands, snout and oral cavities, clinically indistinguishable from those caused by foot-and-mouth disease virus (FMDV), swine vesicular disease virus and vesicular stomatitis virus [4,5].

Although the SVA has been silently circulating in pig herds of the USA since 1988, SVA-caused vesicular disease was not identified in pigs until 2007 in Canada [6]. The second case of SVA infection was identified in the USA in 2010 [7]. At the end of 2014 and the beginning of 2015, increasing outbreaks in weaned and adult pigs were reported in different geographical regions of Brazil [3,8]. Afterwards, SVA emergences were reported in more countries, including China [2], Thailand [9], Vietnam [10] and Colombia [11]. Since its emergence in China in 2015, SVA has affected several provinces, such as Guangdong [12], Fujian [13], Hubei [14] and Heilongjiang [15].

SVA has only one serotype and is a unique species in the genus *Senecavirus* [16]. SVAs are icosahedral virions without envelopes. The diameter of the virus particles is approximately 27 nm. The SVA prototype, SVV-001 strain (Genbank access No.: NC_011349), has an approximately 7300-nt-long genome that contains a 5′ untranslated region (UTR) of 666 nt, followed by a single long open reading frame (ORF) consisting of 6543 nt, encoding a 2181 aa polyprotein. This polyprotein can potentially be cleaved into 12 polypeptides in the standard picornavirus “L–4–3–4 (L–VP4–VP2–VP3–VP1–2A–2B–2C–3A–3B–3C–3D)” layout. The L region encodes the leader protein that can be cleaved from the nascent polyprotein precursor. The P1 region codes for four structural proteins (VP4, VP2, VP3, and VP1): the P1 polypeptide is cleaved by the 3C protease to form VP0, VP3 and VP1, and subsequently a maturation cleavage of VP0 occurs to form the VP2 and the internally located VP4. The P2 and P3 regions encode three (2A, 2B and 2C) and four (3A, 3B, 3C and 3D) nonstructural proteins, respectively. A 3′ UTR of 71 nt is followed by a poly(A) tail of unknown length [17].

Reverse genetics as a platform can be used for genetic manipulation of viral cDNA to construct desired virus phenotype. Different SVA strains have been used to develop reverse genetics systems for studying viral pathogenesis [18], oncolytic activity [19] and recombination mechanism [20]. The SVA CH-LX-01-2016 was an isolate, emerging in Guangdong Province of China in 2016 [21]. Its reverse genetics system has been constructed previously at our laboratory, and proven to be efficient for rescuing recombinant SVAs to express foreign proteins. In this study, we constructed a recombinant SVA (rSVA) that could express an enhanced green fluorescent protein (eGFP) in cells. Its fluorescence-tracked characteristics greatly facilitated both virus neutralization test (VNT) and antiviral assay.

## 2. Materials and Methods

### 2.1. Cell Line, Plasmid and Virus

The T7 RNA polymerase-expressing BHK (BSR-T7/5, or BSR in this study) cells [22] were cultured at 37 °C with 5% CO_2_ in Dulbecco’s modified Eagle’s medium (DMEM), supplemented with 10% fetal bovine serum (FBS) and containing penicillin (100 U/mL), streptomycin (100 µg/mL), amphotericin B (0.25 µg/mL) and G418 (500 µg/mL). The full-length sequence of SVA CH-LX-01-2016 (Genbank access No.: KX751945) genome had been chemically synthesized and subcloned into the pcDNA 3.1 plasmid (Thermo Fisher, Waltham, MA, USA) for construction of a rSVA cDNA clone, which had been proven to be able to rescue the rSVA at our laboratory.

### 2.2. Construction of Plasmid

**Figure 1:**
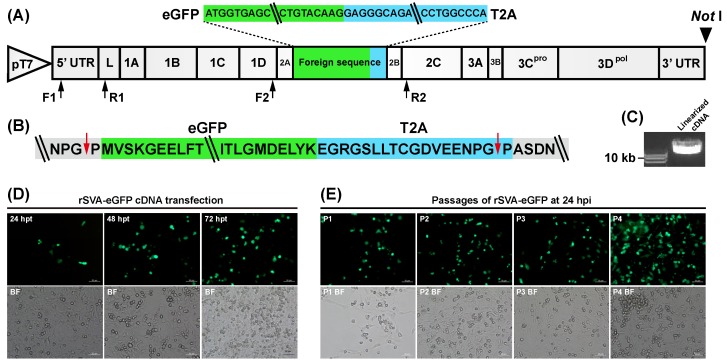
Construction of rSVA-eGFP cDNA clone for rescuing eGFP-expressing recombinant SVA. Schematic representation of rSVA-eGFP cDNA clone (**A**). The eGFP-T2A fusion sequence is inserted between the 2A and 2B genes in the backbone of rSVA cDNA clone, flanked by the T7 promoter and the *Not* I recognition sequences at its 5′ and 3′ ends, respectively. Two pairs of primers, F1/R1 and F2/R2 (black arrow-marked), are designed for RT-PCR analyses on rSVA-eGFP genome and on foreign sequence, respectively. Schematic representation of two self-cleavage sites (red arrow-marked) in the rSVA-eGFP polyprotein (**B**). Agarose gel electrophoresis of *Not* I-linearized rSVA-eGFP cDNA clone (**C**). The rSVA-eGFP cDNA clone-transfected BSR cell monolayers at 24, 48 and 72 hpt (**D**). BSR cell monolayers infected with the rescued rSVA-eGFP at P1, P2, P3 and P4 at 24 hpi (**E**). BF: bright field (the same as following figures).

The rSVA cDNA clone (see Subheading 2.1) was modified to construct another one, rSVA-eGFP cDNA clone (Figure 1A), containing an eGFP ORF (Genbank access No.: KY295913) but without a stop codon at its 3′ end, which was fused with a *Thosea asigna* virus 2A (T2A) sequence (Genbank access No.: JN717245). The eGFP-T2A fusion sequence was inserted between the 2A and the 2B of rSVA cDNA clone using an In-Fusion^®^ Cloning kit (Takara, Dalian, China). The rSVA-eGFP cDNA sequence was flanked by the T7 promoter and the *Not* I recognition sequences at its 5′ and 3′ ends, respectively. In order to enhance the transcription efficiency of T7 promoter, three guanine (G) residues were added to its 3′ end [23].

### 2.3. Rescue of rSVA-eGFP

The rSVA-eGFP cDNA clone was linearized by digestion with *Not* I restriction endonuclease, followed by agarose gel electrophoresis for extraction of the linearized plasmid from gel by a gel extraction kit (TIANGEN, Beijing, China). The purified cDNA was used for transfection of BSR cells using Lipofectamine 2000 (Thermo Fisher, Waltham, MA, USA) according to the manufacturer’s instruction. Briefly, BSR cells were seeded into a 12-well plate 1 d before transfection. Twenty-four hours later, a cell monolayer at 70–90% confluency was transfected with the linearized cDNA clone (1.5 µg/well). Transfected cells were cultured at 37 °C with 5% CO_2_ in DMEM supplemented with 10% FBS, and observed by a fluorescence microscope at 24, 48 and 72 h post transfection (hpt). The BSR cell culture was harvested at 72 hpt, and subjected to one freeze-and-thaw cycle to collect supernatant for five serial blind passages in BSR cells.

### 2.4. Identification of rSVA-eGFP

#### 2.4.1. RT-PCR Analysis

The culture supernatant of rSVA-eGFP at passage 5 (P5) was harvested for extracting viral RNA by a Viral RNA/DNA Extraction Kit (Takara, Dalian, China). The extracted RNA was used as template for RT-PCR analysis using a High Fidelity One Step RT-PCR Kit (Takara, Dalian, China). The forward/reverse primers, F1/R1 (Table 1 and Figure 1A), were designed for amplifying a 693-bp fragment. The RT-PCR reaction underwent 45 °C for 10 min, 94 °C for 2 min and then 30 cycles at 98 °C (10 s), 55 °C (15 s) and 68 °C (10 s). The extracted RNA was simultaneously subjected to PCR analysis as a control using the F1/R1 primer pair. The PCR reaction contained 2 × PrimeSTAR Max Premix (Takara, Dalian, China) and underwent 30 cycles at 98 °C (10 s), 55 °C (5 s) and 72 °C (10 s). RT-PCR and PCR products were detected by agarose gel electrophoresis, followed by Sanger sequencing for analyzing the RT-PCR product.

#### 2.4.2. Indirect Immunofluorescence Assay (IFA) and Ultrathin Section

BSR cells were infected (MOI = 1) with the rSVA-eGFP for 12 h, and then fixed in 4% paraformaldehyde at room temperature for 30 min. After fixation, cells were washed four times with PBS, and then permeated with 0.4% Triton X-100 at room temperature for 30 min. After permeation, cells were washed three times with PBS and blocked in blocking solution (Takara, Dalian, China) at 37 °C for 1 h. Subsequently, cells were incubated with the anti-VP1 monoclonal antibody (MAb, 1: 400 in blocking solution) at 37 °C for 2 h. After incubation with the primary antibody, cells were washed three times with PBS and incubated with the Alexa Fluor^®^ 555 conjugate (Thermo Fisher, Waltham, MA, USA) (1: 250 in blocking solution) at 37 °C for 1 h. Cells were washed three times with PBS, coated with 90% glycerin, and visualized under the fluorescence microscope.

BSR cells were infected (MOI = 1) with the rSVA-eGFP for 12 h, and then collected to prepare ultrathin section for transmission electron microscope (TEM) observation at the Wuhan Servicebio Technology Co., Ltd. (Wuhan, China).

### 2.5. Characterization of rSVA-eGFP

#### 2.5.1. Timeline of Fluorescence Appearance

BSR cells were seeded into ten 35-mm dishes (7 × 10^5^ cells/dish) for culture at 37 °C for 2 h, and then all cell monolayers were inoculated (MOI = 1) with the rSVA-eGFP for further incubation at 37 °C. The dishes were randomly removed from the incubator per hour for observation of green fluorescence until all dishes were removed.

#### 2.5.2. Plaque Assay

Serial 10-fold dilutions were made by mixing 120 µL of rSVA-eGFP with 1080 µL of DMEM. One mL of each dilution was seeded to individual wells of a 6-well plate containing confluent BSR cells. The plate was incubated at 37 °C for 1.5 h before the layer of 1% low melting point agarose (in DMEM with 10% FBS) was added. After 3 d incubation at 37 °C, fluorescent cytopathic effects (CPE) on cell monolayers were observed by the fluorescence microscope. The cell monolayers were fixed in 4% paraformaldehyde and then stained with 0.2% crystal violet. Plaque morphology was observed after washing the plate with tap water, and compared with that of the rSVA.

#### 2.5.3. Growth Curve

Growth kinetics of the P5 rSVA-eGFP were compared with that of the P5 rSVA in vitro. Briefly, BSR cells were plated into four 35-mm dishes (2 × 10^6^ cells/dish) for incubation at 37 °C for 2 h. The rSVA-eGFP was inoculated into all dishes (MOI = 0.001) for incubation at 37 °C for 2 h, and then supernatants were replaced with DMEM for further incubation at 37 °C. At 0, 24, 48, 72 h post infection (hpi), any of dishes was randomly removed from the incubator and subjected to one freeze-and-thaw cycle to collect supernatant for viral titration by 50% tissue culture infective dose (TCID_50_) assay. The viral titer was calculated by the Spearman–Kärber equation [24].

#### 2.5.4. eGFP Stability during Passaging

The rescued rSVA-eGFP was serially passaged in BSR cells fifteen times. Eight BSR cell monolayers were inoculated with the P1, P3, P5, P7, P9, P11, P13 and P15 rSVA-eGFPs, respectively, and then incubated at 37 °C for 24 h. Fluorescence intensities corresponding to their progenies (P2, P4, P6, P8, P10, P12, P14 and P16) were observed under the fluorescence microscope. The culture supernatants at P6, P8, P10, P12, P14 and P16 were harvested to extract viral RNAs for RT-PCR analysis using the F1/R1 primer pair, as described in Subheading 2.4.1. In order to analyze the foreign sequence in the rSVA-eGFP genome, another primer pair (F2/R2, Table 1 and Figure 1A) was designed for amplifying a 1004-bp fragment by RT-PCR using the High Fidelity One Step RT-PCR Kit (Takara, Dalian, China). The RT-PCR reaction underwent 45 °C for 10 min, 94 °C for 2 min and then 30 cycles at 98 °C (10 s), 55 °C (15 s) and 68 °C (10 s). All F1/R1- and F2/R2-amplified products were detected by agarose gel electrophoresis, and the latter was subjected to Sanger sequencing.

### 2.6. rSVA-eGFP-Based VNT

#### 2.6.1. Production of Anti-SVA Polyclonal Antibodies (PAb)

Anti-SVA PAb would be used as a positive control for the VNT. Several female Balb/c mice (Jinan Pengyue Co., Ltd., Jinan, China) were first immunized subcutaneously using formalin-inactivated rSVA supernatant mixed with complete Freund’s adjuvant (Sigma, St. Louis, USA) (*v*/*v*: 1/1, 10^8^ TCID_50_/mouse). After three weeks, vaccinated mice were boosted subcutaneously with formalin-inactivated rSVA supernatant mixed with incomplete Freund’s adjuvant (Sigma, St. Louis, USA) (*v*/*v*: 1/1, 5 × 10^7^ TCID_50_/mouse). After two weeks, blood samples were collected from all mice, and subjected to centrifugation at 5000 g for 10 min to separate antisera. Neutralization titers of anti-rSVA-eGFP sera were assayed in 96-well plates, and those with neutralization titer greater than 640 could be used as positive controls for VNT.

#### 2.6.2. VNT of Porcine Sera

Eight serum samples, collected from SVA-infection-like pigs, were kindly provided by Dr. Bo Ni, China Animal Health and Epidemiology Center, and by Dr. Guimei Li, Qingdao Agricultural University. A standard VNT was carried out in two 96-well plates, as described in literature [25] with a minor modification. The experiment was independently repeated three times. Briefly, the serum was diluted 1:5 after heat-inactivation at 56 °C for 30 min, and then was two-fold serially diluted (1:10 to 1:1280) in DMEM. One hundred µL of rSVA-eGFP (10^3^ TCID_50_/_mL_) was mixed with 100 µL of a given dilution of serum in wells of a 96-well plate, eight replicate wells per dilution. The last three columns were 8-well positive control (positive serum mixed with 100 TCID_50_ rSVA-eGFP), negative control (negative serum mixed with 100 TCID_50_ rSVA-eGFP) and mock (virus- and serum-free DMEM), respectively. Besides these three controls, a series of 8-well rSVA-eGFP controls without serum were arranged in the first four columns of another 96-well plate as follows: 100 TCID_50_/well, 10 TCID_50_/well, 1 TCID_50_/well and 0.1 TCID_50_/well.

All 96-well plates for VNTs of eight sera were incubated at 37 °C for 1.5 h. Cell suspension was added to each well (3 × 10^4^ cells/well), and then all plates were incubated at 37 °C for 48 h for the serum titration. If the rSVA-eGFP was correctly diluted in the rSVA-eGFP control wells, green fluorescence would be emitted from all wells with 100 and 10 TCID_50_/well dilutions, from approximately half the wells with 1 TCID_50_/well dilution, but not from the wells with 0.1 TCID_50_/well dilution. The neutralization titer was the dilution of serum that neutralized rSVA-eGFP in half the wells of a given column. A neutralization titer of ≥ 20 would be regarded as neutralization titer-positive.

### 2.7. rSVA-eGFP-Based Antiviral Assay

#### 2.7.1. Ribavirin

The concentration of ribavirin that reduced the viability of BSR cells to 50% of the control was estimated as the 50% cytotoxic concentration (CC_50_). The MTT assay was used to determine BSR cell viability. Briefly, BSR cells were seeded in a 96-well plate at a density of 2 × 10^4^ cells/well, and incubated at 37 °C for 3 h. Supernatants were replaced with DMEM containing different concentrations of ribavirin (0, 8, 16, 32, 64, 128, 256, 512, 1024 and 2048 μM) (Solarbio, Beijing, China), five replicate wells per dilution. The ribavirin-treated cells were incubated at 37 °C for 24 h. Subsequently, MTT (Sangon, Shanghai, China) solution was added to each well in a final concentration of 0.5 mg/mL, and then the 96-well plate was incubated at 37 °C. After 4 h incubation, supernatants were replaced with dimethyl sulfoxide (DMSO, 100 μL/well) to dissolve violet formazan crystals. The plate was read using a microplate reader at 570 nm wavelength. The CC_50_ value for ribavirin was calculated by nonlinear regression fitting using the GraphPad Prism software (Version 7.0).

Based on the result of cytotoxicity assay, the ribavirin was tested for its antiviral activities at different concentrations. Briefly, BSR cells were seeded in a 24-well plate at a density of 10^6^ cells/well and incubated at 37 °C for 3 h. Subsequently, the cells were inoculated with rSVA-eGFP (MOI = 0.1) and incubated at 37 °C for 1.5 h. Supernatants were replaced with DMEM containing different concentrations of ribavirin (0, 4, 8, 16, 32 and 64 μM). After incubation at 37 °C for 24 h, ribavirin-treated cells were observed by the fluorescence microscope. The 24-well plate was subjected to two freeze-and-thaw cycles to collect supernatants for TCID_50_ assays of rSVA-eGFP. Antiviral activity of ribavirin was expressed as the 50% effective concentration (EC_50_), which was calculated by nonlinear regression fitting using the GraphPad Prism software to determine the drug concentration required to achieve 50% of viral titer reduction [26].

#### 2.7.2. Apigenin

Unlike the water-soluble ribavirin, the apigenin was dissolved in DMSO as its stock solution (25 mg/mL). Therefore, DMSO was arranged in a series of controls at their corresponding concentrations (0%, 0.004%, 0.008%, 0.016%, 0.032%, 0.064% and 0.128%) to those (0, 3.7, 7.4, 14.8, 29.6, 59.2 and 118.4 μM) of apigenin (Solarbio, Beijing, China). The CC_50_ values for apigenin and DMSO were calculated by nonlinear regression fitting using the GraphPad Prism software after the MTT assays as described in Subheading 2.7.1. Antiviral activities of apigenin at different concentrations (0, 3.7, 7.4, 14.8, 29.6, 59.2 and 118.4 μM) were assayed as described in Subheading 2.7.1.

### 2.8. Statistics

Experiments were performed three times with reproducible results. The GraphPad Prism software was used for statistical analysis by two-tailed Student′s *t*-test. The data shown are the means ± standard deviations (SD) of three independent experiments. Statistical significance: * *p* < 0.05, ** *p* < 0.01 and *** *p* < 0.001.

## 3. Results

### 3.1. Construction and Characterization of rSVA-eGFP cDNA Clone

The backbone of rSVA-eGFP cDNA clone was the rSVA (CH-LX-01-2016) cDNA clone. As shown in Figure 1A, the eGFP-T2A fusion sequence was inserted between the 2A and 2B sequences of the rSVA cDNA clone by the In-Fusion^®^ recombineering technology. To facilitate linearization of the recombinant plasmid, the *Not* I recognition sequence was added to the 3′ end of rSVA-eGFP cDNA clone. During translation of the rSVA-eGFP polyprotein, the ribosome initially skipped at the “TNPG↓P” motif of SVA 2A protein, and then did once again at another “TNPG↓P” motif in the T2A protein. Two consecutive events of ribosomal skipping led to releasing an eGFP, flanked by one extra N-terminal proline and a C-terminal T2A cleavage product (Figure 1B).

### 3.2. Rescue and Passaging of rSVA-eGFP

The *Not* I-linearized rSVA-eGFP cDNA clone was subjected to electrophoresis (Figure 1C) and purification for transfection of BSR cells. At 24 hpt, a small number of cells had begun to emit green fluorescence, and the proportion of fluorescence-emitting cells was growing over time (Figure 1D). The cDNA-transfected cells were harvested at 72 hpt, and subjected to one freeze-and-thaw cycle to collect supernatant for serial blind passages, during which the eGFP was always expressed in BSR cells (Figure 1E). Meanwhile, virus-infected cell monolayers showed typical CPEs, characterized by rounding and detachment of cells from flask (Figure 1E, BF).

### 3.3. Identification of rSVA-eGFP

#### 3.3.1. RT-PCR

**Figure 2:**
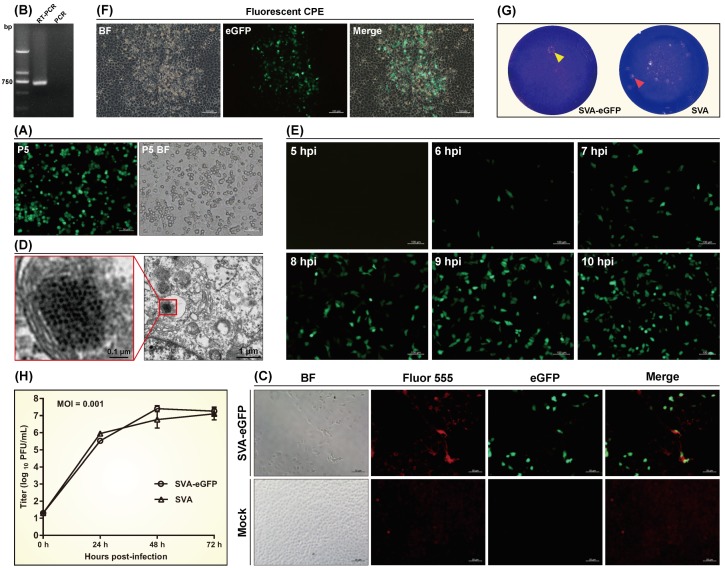
Identification and characterization of rescued rSVA-eGFP. BSR cell monolayer infected with P5 rSVA-eGFP at 24 hpi (**A**). RT-PCR analysis of rSVA-eGFP using the F1/R1 primer pair (**B**). Indirect immunofluorescence assay of rSVA-eGFP-infected BSR cells using the anti-VP1 MAb (primary antibody) and the Alexa Fluor^®^ 555 conjugate (secondary antibody) (**C**). Observation of ultrathin section of rSVA-eGFP-infected BSR cell by transmission electron microscope (**D**). BSR cell monolayers infected with rSVA-eGFP at 5, 6, 7, 8, 9 and 10 hpi (**E**). Fluorescent cytopathic effects (CPE) on cell monolayer infected with rSVA-eGFP at 72 hpi (**F**). Plaque morphology of the rSVA-eGFP (yellow arrowhead-marked), compared with that of the rSVA (red arrowhead-marked) (**G**). Growth curve of the rSVA-eGFP, compared with that of the rSVA (**H**). The data represent the mean ± SD for three independent experiments.

Total RNA was extracted from the P5 rSVA-eGFP at 24 hpi (Figure 2A) and analyzed by RT-PCR to confirm its identity. An expected band of 693-bp amplicon was observed only on the RT-PCR lane by agarose gel electrophoresis (Figure 2B, Lane RT-PCR). The PCR control (Figure 2B, Lane PCR) indicated no potential cDNA contamination affecting the RT-PCR analysis. The Sanger sequencing showed that the P 5-based RT-PCR product was identical to the 693-bp sequence.

#### 3.3.2. IFA

To assess expression of the SVA VP1, the IFA was performed using the VP1 MAb and the Alexa Fluor^®^ 555 conjugate. Bright red fluorescence was visible on the virus-infected cell monolayer but not on the mock cells. Serial treatments (4% paraformaldehyde, 0.4% Triton X-100 and so on) for cells by the IFA did not significantly affect eGFP activity in cells, and most cell contours with red and green fluorescence could be merged together (Figure 2C).

#### 3.3.3. Ultrathin Section

The TEM observation of ultrathin section revealed a typical morphological criterion of picornaviruses [27,28,29] in an rSVA-eGFP-infected cell. Virus particles, unsure whether or not fully matured, appeared as round-, oval- or hexagon-shaped opaque bodies, closely packed together and surrounded by a small mass of light-colored material in a cellular vesicle (Figure 2D). These opaque bodies inside the cell showed an average diameter of 20 nm, less than that (27 nm) of purified SVA virions by CsCl-gradient centrifugation [17].

### 3.4. Characterization of rSVA-eGFP

#### 3.4.1. Timeline of Fluorescence Appearance

The rSVA-eGFP was inoculated into BSR cells (MOI = 1) to observe fluorescence appearance from 0 to 10 hpi. The result showed that virus-infected cells began to emit green fluorescence at 6 hpi. Fluorescence became increasingly bright from 6 hpi to 9 hpi, and was relatively stable after 9 hpi (Figure 2E).

#### 3.4.2. Plaque Assay

After 3 d incubation at 37 °C, a typical CPE with fluorescence was apparent on the cell monolayer (Figure 2F). Plaque morphology of the rSVA-eGFP was further determined by plaque assay. A single plaque was visible on the cell monolayer infected with the rSVA-eGFP at the highest dilution, morphologically similar to those induced by its parental virus (rSVA), but slightly blurred and not easily distinguished from a blue background (Figure 2G).

#### 3.4.3. Growth Curve

At the indicated time points, virus-infected cell cultures were frozen and thawed once to collect supernatant for titration in BSR cells. The growth curve of the P5 rSVA-eGFP was compared with that of the P5 rSVA (Figure 2H). Both viruses, albeit identified as identical initial titers at 0 hpi, showed a slight difference in growth kinetics during 72 h. Their final titers were similar to each other at 72 hpi.

#### 3.4.4. eGFP Stability during Passaging

**Figure 3:**
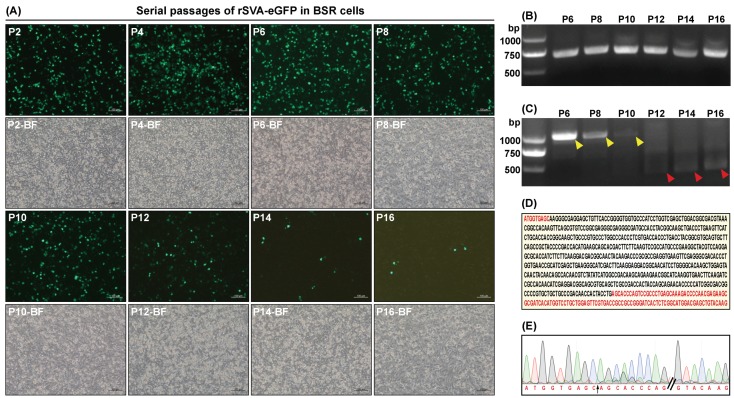
Stability of eGFP expressed by the rSVA-eGFP during serial passaging in BSR cells. Cell monolayers infected with rSVA-eGFP at P2, P4, P6, P8, P10, P12, P14 and P16 at 24 hpi (**A**). RT-PCR analyses of rSVA-eGFP at P6, P8, P10, P12, P14 and P16 using the F1/R1 (**B**) and the F2/R2 (**C**) primer pairs, respectively. Brightness of 1004-bp-specific band (**C**, yellow arrowhead-marked) is gradually decreasing from the P6 to P10, and finally unobservable from the P12 to P16, whereas another band (**C**, red arrowhead-marked) instead gradually becomes bright from the P12 to P16. The ORF sequence of eGFP, from which a 597-bp sequence (**D**, black letters) is deleted from the P12 to P16, and which accordingly retains a 120-bp fragment (**D**, red letters). Sequencing chromatogram of the P16 rSVA-eGFP (**E**). The black arrow illustrates a possible site, where the 597-bp sequence (**D**, black letters) is deleted.

Fluorescence intensity inside virus-infected cells was gradually weakening after the tenth rSVA-eGFP passage. Especially in the P16-infected cell monolayer, there were only sporadic cells that could emit green fluorescence (Figure 3A, P16). The F1/R1-based RT-PCR showed that although the level of eGFP expression was gradually weakening after the tenth passage, the 693-bp-specific product was successfully amplified from all viruses (P6, P8, P10, P12, P14 and P16) (Figure 3B). However, the other RT-PCR analysis using the F2/R2 primer pair revealed that the brightness of 1004-bp-specific band (Figure 3C, yellow arrowhead-marked) was gradually decreasing from the P6 to P10, and finally unobservable from the P12 to P16, whereas another band (Figure 3C, red arrowhead-marked) instead gradually became bright from the P12 to P16.

All six products of the F2/R2-based RT-PCR were subjected to Sanger sequencing, indicating that a 597-bp sequence (Figure 3D, black letters) was deleted from the eGFP ORF from P12 to P16. Consequently, the eGFP ORF only retained a 120-bp fragment (Figure 3D, red letters), out of which nine nucleotides derived from the eGFP 5′ end and the others derived from its 3′ end. The P16 sequencing chromatogram was shown in Figure 3E, in which the black arrow illustrated a possible site, where the 597-bp sequence was deleted.

### 3.5. rSVA-eGFP-Based VNT

**Figure 4:**
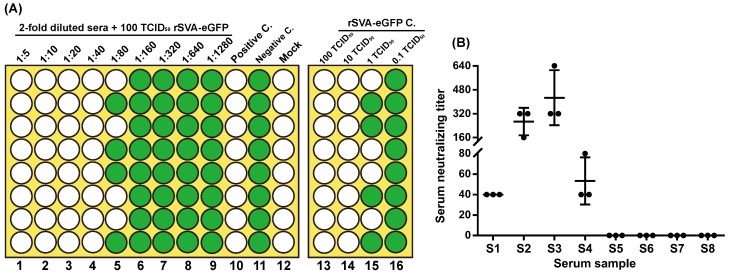
Development of rSVA-eGFP-based virus neutralization test (VNT) for analysis of serum samples. Schematic representation of 96-well plate format designed for performing rSVA-eGFP-based VNT (**A**), which shows a hypothetical result that the neutralization titer is determined to be 80 for a given serum sample. The neutralization titer is the dilution of serum that neutralizes rSVA-eGFP in half the wells of a given column. Green and white wells represent fluorescence-emitting and -free cell monolayers, respectively. C.: Control. VNT result of eight porcine serum samples (**B**), showing that S1, S2, S3 and S4 are neutralization titer-positive; S5, S6, S7 and S8 have no neutralizing activity.

The sera were collected from rSVA-immunized mice for VNT. The result showed that the formalin-inactivated rSVA elicited a high-titer neutralizing antibody response in most mice. Neutralization titers ranged from 320 to 1280, and all pre-immunization sera were negative. Sera with neutralization titer greater than 640 could be used as positive controls for developing the rSVA-eGFP-based VNT in 96-well plates. Figure 4A was a schematic representation of 96-well plate format designed for performing rSVA-eGFP-based VNT. Eight serum samples were collected from pigs with suspected SVA infection, and then analyzed by the developed VNT. The result showed that four samples (S1, S2, S3 and S4) were positive for rSVA-eGFP neutralization, and the others (S5, S6, S7 and S8) had no neutralizing activity (Figure 4B).

### 3.6. Antiviral Assay

#### 3.6.1. Ribavirin

**Figure 5:**
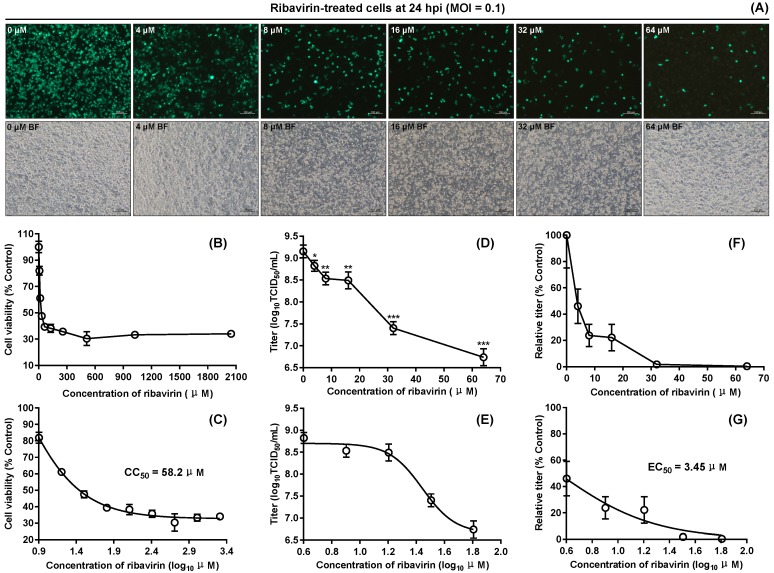
Anti-rSVA-eGFP assay of ribavirin in vitro. Cell monolayers with treatment of ribavirin at different concentrations (0, 4, 8, 16, 32 and 64 μM) at 24 hpi (**A**). Each cell monolayer is observed in a randomly selected field-of-view of fluorescent microscope. Relative viability of BSR cells at 24 h after ribavirin treatment at different concentrations (0, 8, 16, 32, 64, 128, 256, 512, 1024 and 2048 μM) by MTT assay (**B**). The data represent the mean ± SD for five replicates per dilution. The CC_50_ value for ribavirin, calculated by nonlinear regression fitting using the GraphPad Prism software (**C**). Variation in rSVA-eGFP titers at 24 h after ribavirin treatment at different concentrations (0, 4, 8, 16, 32 and 64 μM) (**D**). The data represent the mean ± SD for three independent experiments. *p < 0.05, **p < 0.01 or ***p < 0.001: comparison between the 0 μM group and another one by two-tailed Student′s *t*-test. The data in (**D**) are processed by nonlinear regression fitting using the GraphPad Prism software, and then shown in (**E**). Variation in relative titers at 24 h after ribavirin treatment at different concentrations (0, 4, 8, 16, 32 and 64 μM) (**F**). The data represent the mean ± SD for three independent experiments. The EC_50_ value for ribavirin against rSVA-eGFP, calculated by nonlinear regression fitting using the GraphPad Prism software (**G**).

The effect of ribavirin on BSR cell viability was determined by the MTT assay (Figure 5B). The CC_50_ value was 58.2 μM for the ribavirin (Figure 5C), calculated by nonlinear regression fitting using the GraphPad Prism software. Based on the CC_50_ value, the ribavirin was tested for its antiviral activities at different concentrations. Ribavirin-treated cell monolayers were observed at 24 hpi in a randomly selected field-of-view of fluorescent microscope. As the ribavirin concentration increased, there was a gradual decrease in the proportion of green cells (Figure 5A). All ribavirin-treated cell cultures were titrated by TCID_50_ assay at 24 hpi. Like observation under the fluorescence microscope (Figure 5A), both the absolute titer (Figure 5D,E) and the relative titer (Figure 5F,G) gradually decreased with the increase in ribavirin concentration. The antiviral effect of ribavirin was quantitatively measured by determination of the EC_50_ value, estimated at 3.45 μM (Figure 5G). Effectiveness of an antiviral drug was generally described in terms of the selectivity index (SI, CC_50_/EC_50_), which was 16.87 in this study.

#### 3.6.2. Apigenin

**Figure 6:**
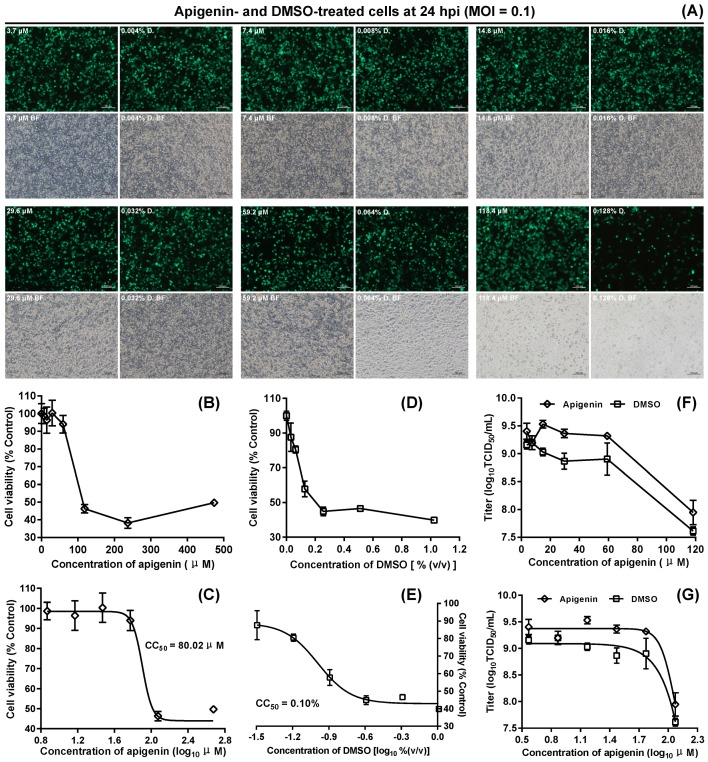
Anti-rSVA-eGFP assay of apigenin in vitro. Apigenin- and DMSO-treated cell monolayers at different concentrations at 24 hpi (**A**). Each cell monolayer is observed in a randomly selected field-of-view of fluorescent microscope. D: DMSO control. Relative viability of BSR cells at 24 h after either apigenin (**B**) or DMSO (**D**) treatment at different concentrations by MTT assay. The data represent the mean ± SD for five replicates per dilution. The CC_50_ value either for apigenin (**C**) or for DMSO (**E**), calculated by nonlinear regression fitting using the GraphPad Prism software. Variations in rSVA-eGFP titers at 24 h after either apigenin or DMSO treatment at different concentrations (**F**). The data represent the mean ± SD for three independent experiments. The data in (**F**) are processed by nonlinear regression fitting using the GraphPad Prism software, and then shown in (**G**).

Apigenin was dissolved in DMSO for preparing its stock solution. Cytotoxicities of apigenin (Figure 6B) and DMSO (Figure 6D) were separately determined in BSR cells by the MTT assay. The CC_50_ values for apigenin and for DMSO were 80.02 μM (Figure 6C) and 0.10% (Figure 6E), respectively, calculated by nonlinear regression fitting after the MTT assay. The apigenin was tested for its antiviral activities at different concentrations, and DMSO was used as controls at corresponding concentrations to those of apigenin. Apigenin-treated cell monolayers were observed at 24 hpi in a randomly selected field-of-view of fluorescent microscope.

However, unlike the ribavirin, the apigenin did not show gradual decreases in the proportion of green cells with serial increases in drug concentration (Figure 6A). Interestingly, the mean size of fluorescence-emitting cells was larger in the 118.4 μM apigenin group than those in the other groups. In comparison with other DMSO controls, the group of 0.128% DMSO revealed a notable reduction in the proportion of green cells (Figure 6A). All apigenin- and DMSO-treated cell cultures were titrated by TCID_50_ assay at 24 hpi, showing that, instead of inhibiting rSVA-eGFP proliferation, all apigenin-treated groups exhibited high viral titers, compared with their DMSO controls (Figure 6F,G). Although the titer decreased dramatically in the 118.4 μM apigenin group, this concentration (118.4 μM) was higher than the CC_50_ value (80.02 μM) of apigenin.

## 4. Discussion

SVA infection was an emerging disease that recently affected numerous pig farms in different provinces of China. The SVA CH-LX-01-2016 was an isolate that had emerged in Guangdong Province of China in 2016 [21]. Its reverse genetics system has been constructed previously at our laboratory, and proven to be efficient for rescuing recombinant SVAs to express foreign proteins, such as luciferase. In this study, we modified the cDNA clone of SVA CH-LX-01-2016 to rescue successfully the eGFP-expressing recombinant virus for facilitating VNT and antiviral assay.

Inserting a foreign gene into the SVA cDNA clone is the first step to rescue a recombinant virus. In this study, the eGFP-T2A fusion sequence was inserted between SVA 2A and 2B genes to construct the rSVA-eGFP cDNA clone (Figure 1A). The T2A peptide can mediate “cleavage” of a polyprotein during its translation in eukaryotic cells [30], accordingly releasing an eGFP-T2A fusion protein that should not obviously affect functions of other SVA proteins. Generally, it was not difficult to construct a recombinant cDNA clone, whereas we found that both the rSVA-eGFP and the rSVA cDNA clones were often unstable in *E.coli* JM109 for an unknown reason. Therefore, recombinant cDNA clones should be extracted immediately from *E.coli* once they have been identified by PCR analysis.

*In vitro* transcription of RNA genome is a common way to rescue recombinant picornaviruses [31,32,33,34,35]. The BSR cell line is permissive to SVA infection, and can provide the T7 RNA polymerase to initiate RNA transcription. Therefore, in this study, BSR cells were directly transfected with the *Not* I-linearized rSVA-eGFP cDNA clone, instead of with in vitro transcription products. The primary transcription product was flanked by “GGG” at its 5′ end and possible *Not* I-digested residues at its 3′ end, both of which did not affect rescue of the rSVA-eGFP, and with replication of the genome RNA in cells, both of which would probably be selectively “ignored” to generate correct 5’ and 3’ termini. When recovered from the cDNA clone, the P0 virus showed a weak adaptability in BSR cells. This cell-adapted ability was gradually improved with serial passaging in vitro. Green fluorescence was always visible during passaging (P0 to P5), and meanwhile the rSVA-eGFP gradually improved itself in growth performance to evolve a high-fitness phenotype in cells.

The identity of rSVA-eGFP was analyzed by RT-PCR, IFA and ultrathin section at the P5. Although the green fluorescence always appeared on cell monolayers during passaging, the RT-PCR analysis was still necessary for detecting recovery of recombinant virus. Meanwhile, PCR as a control was indispensable for eliminating a possible interference of residual cDNA clone. The morphological analysis of SVA was based on the ultrathin section, showing a number of opaque body-like virions, probably mixed with procapsids [36], closely packed together inside a cellular vesicle. Such an intracellular viral morphology was different from that of virions purified from culture supernatant.

Expression of eGFP by the rSVA-eGFP was initially observable at 6 hpi, and was relatively stable after 9 hpi, while the typical CPE would usually appear after 12 hpi. In order to observe complete cell morphology, IFA should be performed during 10 to 12 hpi. In this study, the P5 rSVA-eGFP was chosen for comparing its growth curve with that of its parental virus (P5 rSVA). Based on a quasispecies theory of RNA viruses [37], the same number of viral passages is a prerequisite for comparing their growth kinetics. The eGFP-T2A sequence is a nonstructural gene during rSVA-eGFP replication, and therefore its insertion theoretically affects viral growth kinetics to some extent. Actually, both viruses showed only a slight difference in their growth curves (Figure 2H).

The eGFP-expressing picornaviruses are generally unstable and prone to losing their foreign sequences by a recombination mechanism [20,38]. In this study, the eGFP could be stably expressed in BSR cells up to approximately eight virus passages. Likewise, our previous research showed that a luciferase-expressing SVA could maintain luciferase expression only for nine passages. Owing to the unstable characteristics of eGFP during serial passaging, the rSVA-eGFP was generally restricted to three to five passages for VNT and antiviral assay.

Conventional VNT, albeit time-consuming, is sensitive and specific, thus serving as a prescribed test for international trade of some animal diseases. As a diagnosis of SVA infection, both specificity and sensitivity of the conventional VNT were slightly higher than those of the competitive ELISA [39]. In this study, the rSVA-eGFP-based VNT shortened the incubation period of 96-well plate to 48 h, much less than that of the conventional VNT. Moreover, this novel method was more sensitive and specific than the conventional one using wild-type SVA, because a final VNT result of readout would depend on green fluorescence, instead of CPE. According to our previous study, wild-type SVA-caused CPE was generally too unapparent in some wells to determine a correct result via the conventional VNT. More importantly, since there was only one serotype of SVA worldwide, the novel VNT could be broadly used for clinical diagnosis.

A crucial aspect of the VNT is that the rSVA-eGFP inoculum should be maintained at approximately 100 TCID_50_/well. If significantly greater or less than 100 TCID_50_/well, the final readout for neutralization titer would be correspondingly less or greater than an actual value. Therefore, serial inoculum controls (100, 10, 1 and 0.1 TCID_50_/well) must be arranged in a 96-well plate (Figure 4A, column 13 to 16). The developed VNT was used for detecting eight serum samples, out of which four sera were diagnosed neutralization titer-positive, implying SVA endemic in a given area of China.

A variety of eGFP-tagged viruses have been widely used for antiviral screening [26,40,41,42,43,44]. In this study, the rSVA-eGFP was also proven to be a fast and efficient tool for antiviral assay. The effect of antiviral drug could be visually displayed by variation in eGFP intensities, and then would be quantified by the TCID_50_ assay, by which the decrease in virus titer could be determined 48 h later. Such a time-saving TCID_50_ assay has a great potential in high-throughout screening of anti-SVA drug candidates.

Ribavirin is a synthetic nucleoside analogue that structurally resembles guanosine [45], and can interfere with the synthesis of viral mRNA. It has been demonstrated that ribavirin is a common anti-picornavirus drug with low cytotoxicity [46,47,48,49,50]. Its antiviral effect was generally described in terms of the SI value. A high SI value indicates that a given drug preserves cell viability by reducing the level of pathogenic viruses, while low cytotoxicity is observed [51]. A SI value ≥ 4 is generally considered suitable for an antiviral drug [52]. The SI value was 16.87 for ribavirin in this study, suggesting that it was an effective anti-SVA drug with a relatively safe profile.

The other drug analyzed in this study was apigenin, a plant-derived flavonoid, which was practically insoluble in water, but soluble in DMSO. The apigenin has been demonstrated to have the ability to inhibit replication of some picornaviruses, such as FMDV [53] and enterovirus-71 [54,55]. However, we did not find its anti-SVA effect by the rSVA-eGFP-based antiviral assay. Interestingly, in comparison with their corresponding DMSO controls, the apigenin-treated groups exhibited high virus titers instead (Figure 6F,G).

In conclusion, the rSVA-eGFP was constructed here using the developed platform of reverse genetics. The eGFP could be efficiently expressed in BSR cells but was relatively unstable during serial passaging. Its green fluorescence-tracked characteristics made the rSVA-eGFP-based TCID_50_ assay faster and more accurate than the conventional method did so, thus having a great potential in VNT and antiviral assay. Using the rSVA-eGFP, a total of four serum samples were diagnosed neutralization titer-positive, and the ribavirin was demonstrated to possess anti-SVA activity.

## Figures and Tables

**Table 1:** Two pairs of primers for RT-PCR analyses on enhanced green fluorescent protein-tagged recombinant SVA (rSVA-eGFP).

Primers	Sequences (5′ to 3′)	Length of RT-PCR Product (bp)	
		rSVA-eGFP	rSVA
F1	AGGCACAGAGGAGCAACATCCAA	693	693
R1	ATCGTTCACCGATCTAGGGTATT		
F2	AAAAGCACCGCTGTAAAGCATGT	1004	233
R2	TGTTGAACAGAGTGTACCATCTT		

## References

[1] Wu Q., Zhao X., Chen Y., He X., Zhang G., Ma J. (2016). Complete Genome Sequence of Seneca Valley Virus CH-01-2015 Identified in China. Genome Announc..

[2] Wu Q., Zhao X., Bai Y., Sun B., Xie Q., Ma J. (2017). The First Identification and Complete Genome of Senecavirus A Affecting Pig with Idiopathic Vesicular Disease in China. Transbound. Emerg. Dis..

[3] Leme R.A., Alfieri A.F., Alfieri A.A. (2017). Update on Senecavirus Infection in Pigs. Viruses.

[4] Montiel N., Buckley A., Guo B., Kulshreshtha V., VanGeelen A., Hoang H., Rademacher C., Yoon K.J., Lager K. (2016). Vesicular Disease in 9-Week-Old Pigs Experimentally Infected with Senecavirus A. Emerg. Infect. Dis..

[5] Buckley A., Kulshreshtha V., van Geelen A., Montiel N., Guo B., Yoon K.J., Lager K. (2019). Experimental Seneca Valley virus infection in market-weight gilts. Vet. Microbiol..

[6] Pasma T., Davidson S., Shaw S.L. (2008). Idiopathic vesicular disease in swine in Manitoba. Can. Vet. J..

[7] Singh K., Corner S., Clark S., Scherba G., Fredrickson R. (2012). Seneca Valley Virus and Vesicular Lesions in a Pig with Idiopathic Vesicular Disease. J. Vet. Sci. Technol..

[8] Leme R.A., Zotti E., Alcantara B.K., Oliveira M.V., Freitas L.A., Alfieri A.F., Alfieri A.A. (2015). Senecavirus A: An Emerging Vesicular Infection in Brazilian Pig Herds. Transbound. Emerg. Dis..

[9] Saeng-Chuto K., Rodtian P., Temeeyasen G., Wegner M., Nilubol D. (2018). The first detection of Senecavirus A in pigs in Thailand, 2016. Transbound. Emerg. Dis..

[10] Arzt J., Bertram M.R., Vu L.T., Pauszek S.J., Hartwig E.J., Smoliga G.R., Palinski R., Stenfeldt C., Fish I.H., Hoang B.H. (2019). First Detection and Genome Sequence of Senecavirus A in Vietnam. Microbiol. Resour. Announc..

[11] Sun D., Vannucci F., Knutson T.P., Corzo C., Marthaler D.G. (2017). Emergence and whole-genome sequence of Senecavirus A in Colombia. Transbound. Emerg. Dis..

[12] Sun Y., Cheng J., Wu R.T., Wu Z.X., Chen J.W., Luo Y., Xie Q.M., Ma J.Y. (2018). Phylogenetic and Genome Analysis of 17 Novel Senecavirus A Isolates in Guangdong Province, 2017. Front. Vet. Sci..

[13] Zhu Z., Yang F., Chen P., Liu H., Cao W., Zhang K., Liu X., Zheng H. (2017). Emergence of novel Seneca Valley virus strains in China, 2017. Transbound. Emerg. Dis..

[14] Qian S., Fan W., Qian P., Chen H., Li X. (2016). Isolation and full-genome sequencing of Seneca Valley virus in piglets from China, 2016. Virol. J..

[15] Wang H., Li C., Zhao B., Yuan T., Yang D., Zhou G., Yu L. (2017). Complete genome sequence and phylogenetic analysis of Senecavirus A isolated in Northeast China in 2016. Arch. Virol..

[16] Zhang X., Zhu Z., Yang F., Cao W., Tian H., Zhang K., Zheng H., Liu X. (2018). Review of Seneca Valley Virus: A Call for Increased Surveillance and Research. Front. Microbiol..

[17] Hales L.M., Knowles N.J., Reddy P.S., Xu L., Hay C., Hallenbeck P.L. (2008). Complete genome sequence analysis of Seneca Valley virus-001, a novel oncolytic picornavirus. J. Gen. Virol..

[18] Chen Z., Yuan F., Li Y., Shang P., Schroeder R., Lechtenberg K., Henningson J., Hause B., Bai J., Rowland R.R.R. (2016). Construction and characterization of a full-length cDNA infectious clone of emerging porcine Senecavirus A. Virology.

[19] Poirier J.T., Reddy P.S., Idamakanti N., Li S.S., Stump K.L., Burroughs K.D., Hallenbeck P.L., Rudin C.M. (2012). Characterization of a full-length infectious cDNA clone and a GFP reporter derivative of the oncolytic picornavirus SVV-001. J. Gen. Virol..

[20] Li C., Wang H., Shi J., Yang D., Zhou G., Chang J., Cameron C.E., Woodman A., Yu L. (2019). Senecavirus-Specific Recombination Assays Reveal the Intimate Link between Polymerase Fidelity and RNA Recombination. J. Virol..

[21] Zhao X., Wu Q., Bai Y., Chen G., Zhou L., Wu Z., Li Y., Zhou W., Yang H., Ma J. (2017). Phylogenetic and genome analysis of seven senecavirus A isolates in China. Transbound. Emerg. Dis..

[22] Buchholz U.J., Finke S., Conzelmann K.K. (1999). Generation of bovine respiratory syncytial virus (BRSV) from cDNA: BRSV NS2 is not essential for virus replication in tissue culture, and the human RSV leader region acts as a functional BRSV genome promoter. J. Virol..

[23] Martin C.T., Muller D.K., Coleman J.E. (1988). Processivity in early stages of transcription by T7 RNA polymerase. Biochemistry.

[24] Finney D.J. (1952). Statistical Method in Biological Assay.

[25] OIE (2018). Manual of Diagnostic Tests and Vaccines for Terrestrial Animals (Chapter 3.7.9.).

[26] Li X., Zhang H., Zhang Y., Li J., Wang Z., Deng C., Jardim A.C.G., Terzian A.C.B., Nogueira M.L., Zhang B. (2019). Development of a rapid antiviral screening assay based on eGFP reporter virus of Mayaro virus. Antivir. Res..

[27] Chao Y.-C., Young S., Kim K.S. (1986). Characterization of a picornavirus isolated from Pseudoplusia includens (Lepidoptera: Noctuidae). J. Invertebr. Pathol..

[28] Sjostrand F.S., Polson A. (1958). Macrocrystalline patterns of closely packed poliovirus particles in ultrathin sections. J. Ultrastruct. Res..

[29] Peterson J.E., Studdert M.J. (1970). Feline picornavirus. Structure of the virus and electron microscopic observations on infected cell cultures. Arch. Gesamte Virusforsch..

[30] Liu Z., Chen O., Wall J.B.J., Zheng M., Zhou Y., Wang L., Ruth Vaseghi H., Qian L., Liu J. (2017). Systematic comparison of 2A peptides for cloning multi-genes in a polycistronic vector. Sci. Rep..

[31] Flather D., Cathcart A.L., Cruz C., Baggs E., Ngo T., Gershon P.D., Semler B.L. (2016). Generation of Recombinant Polioviruses Harboring RNA Affinity Tags in the 5’ and 3’ Noncoding Regions of Genomic RNAs. Viruses.

[32] Han M., Rajput C., Hinde J.L., Wu Q., Lei J., Ishikawa T., Bentley J.K., Hershenson M.B. (2018). Construction of a recombinant rhinovirus accommodating fluorescent marker expression. Influenza Other Respir. Viruses.

[33] Rieder E., Henry T., Duque H., Baxt B. (2005). Analysis of a foot-and-mouth disease virus type A24 isolate containing an SGD receptor recognition site in vitro and its pathogenesis in cattle. J. Virol..

[34] Rajasekhar R., Hosamani M., Basagoudanavar S.H., Sreenivasa B.P., Tamil Selvan R.P., Saravanan P., Venkataramanan R. (2013). Rescue of infective virus from a genome-length cDNA clone of the FMDV serotype O (IND-R2/75) vaccine strain and its characterization. Res. Vet. Sci..

[35] Shang B., Deng C., Ye H., Xu W., Yuan Z., Shi P.Y., Zhang B. (2013). Development and characterization of a stable eGFP enterovirus 71 for antiviral screening. Antivir. Res..

[36] Strauss M., Jayawardena N., Sun E., Easingwood R.A., Burga L.N., Bostina M. (2018). Cryo-Electron Microscopy Structure of Seneca Valley Virus Procapsid. J. Virol..

[37] Liu F., Wu X., Li L., Zou Y., Liu S., Wang Z. (2016). Evolutionary characteristics of morbilliviruses during serial passages in vitro: Gradual attenuation of virus virulence. Comp. Immunol. Microbiol. Infect. Dis.

[38] Xiao Y., Rouzine I.M., Bianco S., Acevedo A., Goldstein E.F., Farkov M., Brodsky L., Andino R. (2016). RNA Recombination Enhances Adaptability and Is Required for Virus Spread and Virulence. Cell Host Microbe.

[39] Goolia M., Vannucci F., Yang M., Patnayak D., Babiuk S., Nfon C.K. (2017). Validation of a competitive ELISA and a virus neutralization test for the detection and confirmation of antibodies to Senecavirus A in swine sera. J. Vet. Diagn. Investig..

[40] Lo M.K., Nichol S.T., Spiropoulou C.F. (2014). Evaluation of luciferase and GFP-expressing Nipah viruses for rapid quantitative antiviral screening. Antivir. Res..

[41] Fan Z.C., Bird R.C. (2012). Development of a reporter bovine viral diarrhea virus and initial evaluation of its application for high throughput antiviral drug screening. J. Virol. Methods.

[42] Marschall M., Freitag M., Weiler S., Sorg G., Stamminger T. (2000). Recombinant green fluorescent protein-expressing human cytomegalovirus as a tool for screening antiviral agents. Antimicrob. Agents Chemother..

[43] Kim J.I., Park S., Lee I., Lee S., Shin S., Won Y., Hwang M.W., Bae J.Y., Heo J., Hyun H.E. (2012). GFP-expressing influenza A virus for evaluation of the efficacy of antiviral agents. J. Microbiol..

[44] Cai Y., Iwasaki M., Beitzel B.F., Yu S., Postnikova E.N., Cubitt B., DeWald L.E., Radoshitzky S.R., Bollinger L., Jahrling P.B. (2018). Recombinant Lassa Virus Expressing Green Fluorescent Protein as a Tool for High-Throughput Drug Screens and Neutralizing Antibody Assays. Viruses.

[45] Shiffman M.L. (2009). What future for ribavirin?. Liver Int..

[46] Ruuskanen O., Waris M., Kainulainen L. (2014). Treatment of persistent rhinovirus infection with pegylated interferon alpha2a and ribavirin in patients with hypogammaglobulinemia. Clin. Infect. Dis..

[47] Smee D.F., Evans W.J., Nicolaou K.C., Tarbet E.B., Day C.W. (2016). Susceptibilities of enterovirus D68, enterovirus 71, and rhinovirus 87 strains to various antiviral compounds. Antivir. Res..

[48] Li Z.H., Li C.M., Ling P., Shen F.H., Chen S.H., Liu C.C., Yu C.K. (2008). Ribavirin reduces mortality in enterovirus 71-infected mice by decreasing viral replication. J. Infect. Dis..

[49] Kang H., Kim C., Kim D.E., Song J.H., Choi M., Choi K., Kang M., Lee K., Kim H.S., Shin J.S. (2015). Synergistic antiviral activity of gemcitabine and ribavirin against enteroviruses. Antivir. Res..

[50] Choi J.H., Jeong K., Kim S.M., Ko M.K., You S.H., Lyoo Y.S., Kim B., Ku J.M., Park J.H. (2018). Synergistic effect of ribavirin and vaccine for protection during early infection stage of foot-and-mouth disease. J. Vet. Sci..

[51] Jeong K.W., Lee J.H., Park S.M., Choi J.H., Jeong D.Y., Choi D.H., Nam Y., Park J.H., Lee K.N., Kim S.M. (2015). Synthesis and in-vitro evaluation of 2-amino-4-arylthiazole as inhibitor of 3D polymerase against foot-and-mouth disease (FMD). Eur. J. Med. Chem..

[52] Yao C., Xi C., Hu K., Gao W., Cai X., Qin J., Lv S., Du C., Wei Y. (2018). Inhibition of enterovirus 71 replication and viral 3C protease by quercetin. Virol. J..

[53] Qian S., Fan W., Qian P., Zhang D., Wei Y., Chen H., Li X. (2015). Apigenin restricts FMDV infection and inhibits viral IRES driven translational activity. Viruses.

[54] Zhang W., Qiao H., Lv Y., Wang J., Chen X., Hou Y., Tan R., Li E. (2014). Apigenin inhibits enterovirus-71 infection by disrupting viral RNA association with trans-acting factors. PLoS ONE.

[55] Lv X., Qiu M., Chen D., Zheng N., Jin Y., Wu Z. (2014). Apigenin inhibits enterovirus 71 replication through suppressing viral IRES activity and modulating cellular JNK pathway. Antivir. Res..

